# Creating Blood Analogs to Mimic Steady-State Non-Newtonian Shear-Thinning Characteristics Under Various Thermal Conditions

**DOI:** 10.3390/bioengineering12070758

**Published:** 2025-07-12

**Authors:** Hang Yi, Alexander Wang, Christopher Wang, Jared Chong, Chungyiu Ma, Luke Bramlage, Bryan Ludwig, Zifeng Yang

**Affiliations:** 1 Department of Mechanical and Materials Engineering, Wright State University, 3640 Colonel Glenn Hwy., Dayton, OH 45435, USA; 2 Centerville High School, 500 E Franklin St, Centerville, OH 45459, USA; 3 St. Andrew’s College, Aurora, ON L4G 3H7, Canada; 4 Clinical Neuroscience Institute, Premier Health–Miami Valley Hospital, Dayton, OH 45409, USA; 5 Boonshoft School of Medicine, Wright State University, 3640 Colonel Glenn Hwy., Dayton, OH 45435, USA

**Keywords:** blood analog, steady-state, non-Newtonian, shear-thinning, temperature

## Abstract

Blood analogs are widely employed in in vitro experiments such as particle image velocity (PIV) to secure hemodynamics, assisting pathophysiological diagnoses of neurovascular and cardiovascular diseases, as well as pre-surgical planning and intraoperative orientation. To obtain accurate physical parameters, which are critical for diagnosis and treatment, blood analogs should exhibit realistic non-Newtonian shear-thinning features. In this study, two types of blood analogs working under room temperature (293.15 K) were created to mimic the steady-state shear-thinning features of blood over a temperature range of 295 to 312 K and a shear range of 1~250 s^−1^ at a hematocrit of ~40%. Type I was a general-purpose analog composed of deionized (DI) water and xanthan gum (XG) powder, while Type II was specially designed for PIV tests, incorporating DI water, XG, and fluorescent microspheres. By minimizing the root mean square deviation between generated blood analogs and an established viscosity model, formulas for both blood analogs were successfully derived for the designated temperatures. The results showed that both blood analogs could replicate the shear-thinning viscosities of real blood, with the averaged relative discrepancy < 5%. Additionally, a strong linear correlation was observed between body temperature and XG concentration in both blood analogs (coefficient of determination > 0.96): for Type I, 295–312 K correlates with 140–520 ppm, and for Type II, 295–315 K correlates with 200–560 ppm. This work bridges the gap between idealized steady-state non-Newtonian viscosity models of blood and the complexities of real-world physiological conditions, offering a versatile platform for advancing particle image velocimetry tests and hemodynamics modeling, optimizing therapeutic interventions, and enhancing biomedical technologies in temperature-sensitive environments.

## 1. Introduction

Blood is a thixo-elasto-visco-plastic (TEVP) biofluid, meaning that it exhibits shear-thinning, viscoelastic, and thixotropic properties [1]. Notably, its non-Newtonian shear-thinning behavior, that is, its viscosity, decreases as the shear rate increases [2,3], significantly influence hemodynamics in both neurovascular and cardiovascular systems, affecting physiological and pathological diagnoses in vitro and/or in silico from hemodynamic perspectives [4,5]. Additionally, blood viscosity is temperature-dependent, with variations in temperature having a substantial impact on the blood viscosity and hemodynamics within vessels [5].

To replicate realistic blood rheological shear-thinning behavior, transparent blood analogs are often used in vitro, particularly in 3D-printed vascular models, pathophysiology diagnoses, and for the validation of hemodynamic models [6,7]. For example, blood analogs provide realistic non-Newtonian viscosity features at low cost to help researchers evaluate pump efficiency, hemocompatibility, and shear stress effects in ventricular assistance devices [8]. With seeded particles, blood analogs are employed to investigate the shear-induced blood damage of vascular diseases and thrombus formation [5,9].

Traditional Newtonian fluids (e.g., water or glycerol solutions), which assume a constant blood viscosity of approximately 3.0~4.0 mPa·s, fail to capture critical hemodynamic characteristics due to shear rate changes such as instantaneous wall shear stress, the oscillatory shear index, and relative residence time, thus limiting their effectiveness in realistic simulations of blood flow [5,10,11,12,13]. Therefore, the development of non-Newtonian blood analogs that accurately replicate the rheological shear-thinning behavior of human blood under variable temperature conditions is crucial for advancing biomedical research, medical device development, educational demonstrations, and hemodynamic modeling.

Realistic blood analogs must not only exhibit non-Newtonian shear-thinning behavior but also account for the effects of temperature variations. The analog must behave similarly to real blood across a broad range of thermal conditions. For instance, hypothermia (body temperature < 310.15 K) increases blood viscosity and alters its shear-thinning behavior, potentially exacerbating circulatory complications [14], while hyperthermia (body temperature > 310.15 K) decreases viscosity and changes its viscoelastic features [15,16]. These temperature-induced changes have important implications for applications such as cardiopulmonary bypass systems [17], cryopreservation protocols [18], and thermal ablation therapies [19]. Despite this, most existing blood analogs do not consider temperature as a variable, leading to oversimplified experimental or computational models [10,20,21,22,23].

To address knowledge gaps in non-Newtonian blood analogs with shear-thinning properties, making them suitable for in vitro experiments under various thermal conditions, this work presents the design and synthesis of two types (Type I and Type II) of non-Newtonian blood analogs working under room temperature while mimicking human blood’s steady-state rheological properties across a clinically relevant temperature range of 295–312 K [24], at a hematocrit of ~40%. The capabilities of this blood analog, working at room temperature, could simplify in vitro experimental setups significantly by removing the need for a temperature control system. Type I was intended for general use, i.e., where velocity field visualizations/measurements are not required, while Type II was specifically designed for particle image velocimetry (PIV) measurements to investigate hemodynamics in vascular models. Both analogs were based on a solution of 800 ppm xanthan gum (XG), composed of XG power deionized (DI) water, which can exhibit non-Newtonian shear-thinning properties similar to those of real blood [25,26]. It should be mentioned that blood elastic properties (solid-like) under extremely low shear rates were not considered in this study due to the limited capability of the instruments used. In addition, viscosity fluctuation in the transient rheological environment—mainly due to blood cell filterability and erythrocyte aggregation—was not explicitly considered in this study as it has nearly no impact on viscosity at high shear rates (>1 s^−1^) [1,3,27]; thus, such a limitation was expected to have a minimal effect on the objective of this study, i.e., to generate blood analogs to mimic blood steady-state non-Newtonian shear-thinning properties within the shear rate range of 1–250 s^−1^.

Specifically, to create Type I blood analogs, 800 ppm XG solution was diluted with DI water to obtain concentrations ranging from 120 ppm to 600 ppm, in 20 ppm increments. Type II blood analogs were created similarly, but with the addition of 450 ppm fluorescent red polyethylene microspheres (FRPM). The fluorescent light from the particles due to laser excitation enables velocity profiles to be captured in PIV tests. Progress in this research could assist scientists and engineers in the blood and hemodynamics research community to (1) test medical devices (e.g., stents, catheters, pumps) under physiologically realistic temperature scenarios using PIV to validate computational fluid dynamics models, (2) study disease mechanisms (e.g., thrombosis, atherosclerosis, cerebral aneurysms) influenced by temperature gradients, (3) validate computational models that incorporate thermal effects on blood flows, and (4) conduct surgical training and education with transparent blood models using low-lost blood alternatives.

## 2. Materials and Methods

### 2.1. Developing a Shear-Rate and Temperature-Dependent Non-Newtonian Blood Viscosity Model

The generated Type I and Type II blood analogs were compared with our previously developed shear-rate and temperature-dependent non-Newtonian blood viscosity model [5], which has been employed by many other research groups. Details of the data collection and analysis process can be accessed in our previous publication [5]. However, to ensure that the present paper is self-contained, we present our deduction process here to assist the blood analog research community in better understanding the intrinsic coupling mechanisms between shear-thinning behavior and thermal effects regarding blood viscosity [5], that is:

(1) $$\mu =a{\dot{\gamma }}^{b-1}e^{\left(\alpha \left(\frac{1}{T}-\frac{1}{\delta T+\varepsilon }\right)+\beta T\right)},\;T[K]\in [\sim 295,\;\sim 310]$$

In Equation (1), μ is blood viscosity, a is the consistency index equal to $2.05\times 10^{12}$ $mPa\cdot s^{b}$, b is the power-law index registered as 0.685, and T is the blood temperature in K. In addition, α, β, δ, and ε are temperature index constants equating to 4945.4 K, −0.083 $K^{-1}$, 1.53 and −162.22 K, respectively. The rest of this section shows the details of the developing process. The process of data collection can be accessed in our previous publication [5].

In general, the non-Newtonian blood viscosity model can be expressed by a power-law equation [28]:

(2) $$\mu =a{\dot{\gamma }}^{b-1}$$

**Figure 1:**
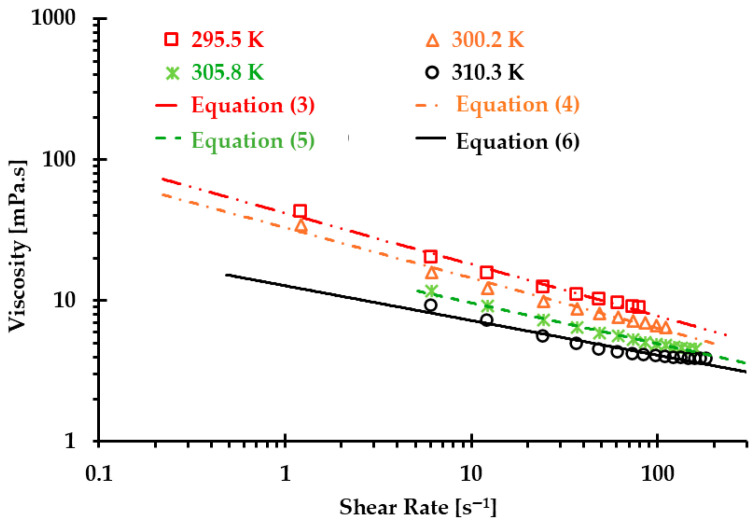
Relationships between experiments and regressed Equations (2)–(6) to predict blood viscosity under temperatures of 295.5, 300.2, 305.8, and 310.3 K.

The viscosity of real blood was measured using an IKA ROTAVISC lo-vi viscometer (IKA-Werke GmbH & Co. KG, Staufen, Germany) under four designated temperatures [5]; the measured viscosities under the corresponding temperatures are shown in Figure 1. Integrating the generalized power-law viscosity model (i.e., Equation (2)) with the collected experimental data, four non-Newtonian power-law viscosity models can be regressed under the corresponding four temperatures. Specifically, the equations are as follows:

(3) $$\mu_{\begin{array}{c} T=295.5\;K\\ \; \end{array}}={a_{1}\dot{\gamma }}^{b_{1}-1},a_{1}=41.927\times 10^{-3}Pa\cdot s^{b_{1}},b_{1}=0.635$$

(4) $$\mu_{\begin{array}{c} T=300.2\;K\\ \; \end{array}}={a_{2}\dot{\gamma }}^{b_{2}-1},a_{2}=32.948\times 10^{-3}Pa\cdot s^{b_{2}},b_{2}=0.643$$

(5) $$\mu_{\begin{array}{c} T=305.8\;K\\ \; \end{array}}={a_{3}\dot{\gamma }}^{b_{3}-1},a_{3}=18.817\times 10^{-3}Pa\cdot s^{b_{3}},b_{3}=0.710$$

(6) $$\mu_{\begin{array}{c} T=310.3\;K\\ \; \end{array}}={a_{4}\dot{\gamma }}^{b_{4}-1},a_{4}=12.763\times 10^{-3}Pa\cdot s^{b_{4}},b_{4}=0.753$$

Correlation coefficients R for the regressed Equations (3)–(6) are 0.975, 0.995, 0.990, and 0.995 under temperatures of 295.5, 300.2, 305.8, and 310.3 K, respectively (see Figure 1).

Considering thermal influences on blood viscosity, it was assumed that the blood viscosity model should consist of two variables, i.e., the shear rate term $\eta \left(\dot{\gamma }\right)$ and the temperature index $H\left(T\right)$, in which $H\left(T\right)$ is based on the Arrhenius law [29,30,31,32]. The equation was expressed as

(7a) $$\mu_{\left(\dot{\gamma },T\right)}=\eta \left(\dot{\gamma }\right)H\left(T\right)$$

(7b) $$\eta \left(\dot{\gamma }\right)=a{\dot{\gamma }}^{b-1}$$

In Equation (7b), b was calculated by averaging the consistency indexes in Equations (3)–(6), i.e.:

$$b=\frac{b_{1}+b_{2}+b_{3}+b_{4}}{4}=0.685$$

**Figure 2:**
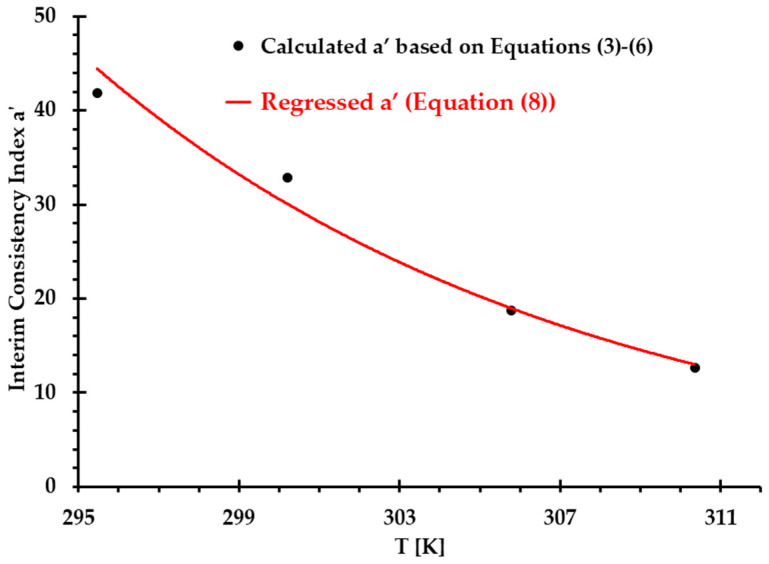
Comparisons of interim consistency index $a^{\prime }$ between Equations (3)–(6) and regressed Equation (8) under designated temperatures.

To find consistency index a in Equation (7b), an interim consistency index $a^{\prime }$ was introduced in this study to present its relationship with temperature (see Figure 2). Using the exponential regressed strategy, $a^{\prime }$ was regressed as a power-law form, that is:

(8) $$a^{\prime }=ae^{-0.083T},a=2.05\times 10^{12}mPa\cdot s^{b}.$$

In Figure 2, the correlation coefficient R of Equation (8) for the interim consistency index $a^{\prime }$ is 0.987 under measured temperatures from 295 to 310 K.

Then, taking the natural logarithm on both sides of Equation (7a) yields:

(9) $$\ln\left(\mu_{\left(\dot{\gamma },T\right)}\right)=\ln\left(\eta \left(\dot{\gamma }\right)\right)+\ln\left(H\left(T\right)\right)$$

Since $\eta \left(\dot{\gamma }\right)$ is a shear rate dependent variable solely, a plot of $\ln\left(\mu_{\left(\dot{\gamma },T\right)}\right)$ vs. $\ln\left(H\left(T\right)\right)$ can be obtained using two different temperatures ($T_{m}$ and $T_{n}$, where m and n represent two different thermal data points, m = 1, 2, 3, and n = 2, 3, 4) under the same shear rate $\dot{\gamma }$, as follows:

(10) $$\ln\left(\mu_{\left(\dot{\gamma },T_{m}\right)}\right)-\ln\left(\mu_{\left(\dot{\gamma },T_{n}\right)}\right)=\ln\left(H\left(T_{m}\right)\right)-\ln\left(H\left(T_{n}\right)\right)$$

Based on Arrhenius law and integrating $e^{-0.083T}$ (i.e., $e^{\beta T}$) into Equation (8), we can define $H\left(T\right)$ as:

(11) $$H\left(T\right)=e^{\alpha \left(\frac{1}{T}-\frac{1}{T_{Ref.}}\right)+\beta T}$$

In Equation (11), α is a temperature index constant related to the activation energy, and β is another temperature index constant registered as −0.083 $K^{-1}$ (see Equation (8)). Additionally, $T_{Ref.}$ is an introduced reference temperature. By integrating Equation (11), Equation (10) can be rewritten as follows:

(12) $$\ln\left(\mu_{\left(\dot{\gamma },T_{m}\right)}\right)-\ln\left(\mu_{\left(\dot{\gamma },T_{n}\right)}\right)-\beta (T_{m}-T_{n})=\alpha \left({\left(\frac{1}{T}-\frac{1}{T_{Ref.}}\right)}_{T=T_{m}}-{\left(\frac{1}{T}-\frac{1}{T_{Ref.}}\right)}_{T=T_{n}}\right)$$

Temporarily, $T_{Ref.}$ is assumed as a constant value which will be further corrected after the temperature index α has been decided. Thus, Equation (12) can be simplified as:

(13) $$\ln\left(\mu_{\left(\dot{\gamma },T_{m}\right)}\right)-\ln\left(\mu_{\left(\dot{\gamma },T_{n}\right)}\right)-\beta (T_{m}-T_{n})=\alpha \left(\frac{1}{T_{m}}-\frac{1}{T_{n}}\right)$$

Further, α can be decided by Equation (14), that is:

(14) $$\ln\left(\mu_{\left(\dot{\gamma },T_{m}\right)}\right)-\ln\left(\mu_{\left(\dot{\gamma },T_{n}\right)}\right)-\beta (T_{m}-T_{n})=\alpha \left(\frac{1}{T_{m}}-\frac{1}{T_{n}}\right)$$

To facilitate the calculations and expressions, we define the following:

(15) $$∆T_{m-n}=\frac{1}{T_{m}}-\frac{1}{T_{n}}$$

By integrating the experimental data shown in Figure 1 and Equation (8), α can be determined with the corresponding shear rate $\dot{\gamma }$, as shown in Table 1.

Averaging the values in Table 1, the temperature index of α can be obtained, i.e.,

α=4945.4 K

The next step was to regress $T_{Ref.}$ in Equation (11). By integrating Equations (7a,b), (8), and (11), we obtained:

(16) $$T_{Ref.}=\frac{1}{\frac{1}{T}-\frac{1}{\alpha }\left(\ln\left(\mu_{\left(\dot{\gamma },T\right)}\right)-ln\left(a{\dot{\gamma }}^{b-1}\right)+0.083T\right)}$$

Using the viscosities and shear rates under corresponding temperatures, the calculated results of $T_{Ref.}$ based on Equation (16) are exhibited in Table 2. Averaging the $T_{Ref.}$ values under the corresponding four thermal conditions, we obtained the following:

$${T_{Ref.}}_{T=295.5\;K}=291.0679\;K\;{T_{Ref.}}_{T=300.2\;K}=298.5440\;K$$

$${T_{Ref.}}_{T=305.8\;K}=306.9594\;K\;{T_{Ref.}}_{T=310.3\;K}=313.9530\;K$$

**Figure 3:**
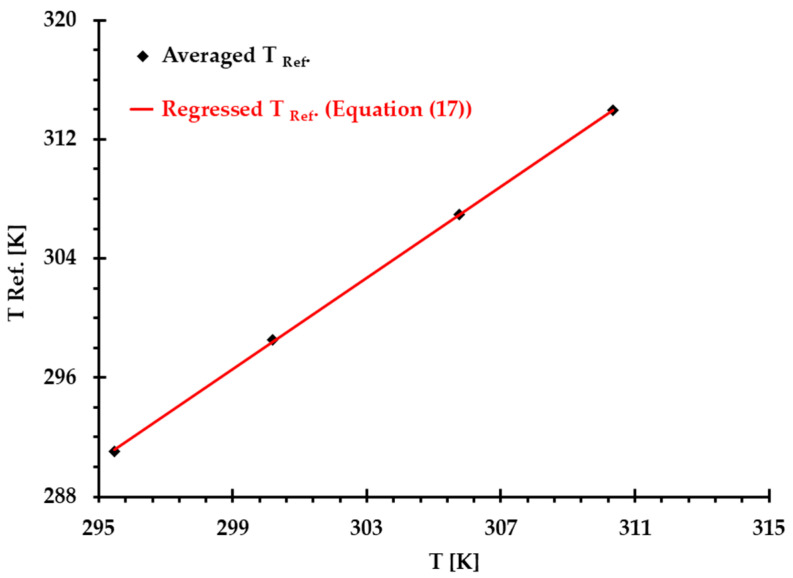
Comparisons of introduced reference temperature $T_{Ref.}$ based on the averaged values (see Table 2) and the values calculated using Equation (17) under and four thermal conditions, where correlation coefficient R=0.999.

By averaging the calculated $T_{Ref.}$ in Table 2 and employing linear regression method, the relationship between reference temperature $T_{Ref.}$ and T (see Figure 3) can be expressed by

(17) $$T_{Ref.}=\delta T+\varepsilon$$

where δ and ε are temperature related constants, i.e., 1.53 and −162.22 K, respectively.

**Figure 4:**
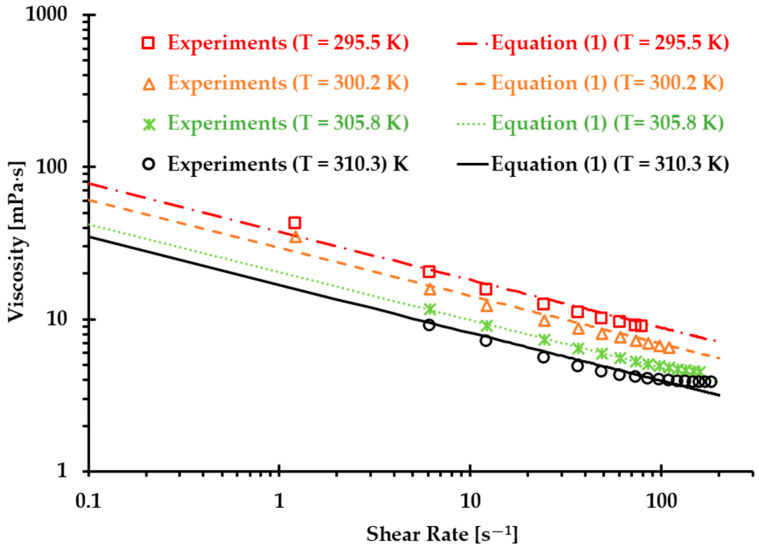
Comparisons between the experimental data and the developed non-Newtonian blood viscosity model (Equation (1)) [5].

Finally, we can obtain the non-Newtonian blood model under various thermal conditions shown in Equation (1). Figure 4 shows the correlation coefficients R between Equation (1) [5] and experimental measurements, which were 0.99, 0.99, 1.00, and 0.98 at T = 310.3, 305.8, 300.2, and 295.5 K, respectively. The results indicated that the developed non-Newtonian model successfully presented blood non-Newtonian shear-thinning properties under various thermal conditions. Figure 4 indicates that blood presents shear-thinning non-Newtonian features, namely, as viscosity decreases, the shear rate increases under all four designated temperatures. In addition, blood viscosity manifests non-isothermal properties, that is, as temperature increases, the viscosity decreases gradually under the same shear rate. In this study, the developed Equation (1) was employed as the baseline equation to estimate the accuracy of the generated Type I and Type II blood analogs by minimizing the root mean square deviation (RMSD).

However, it needs to be mentioned that the data for plotting the non-Newtonian viscosity model (i.e., Equation (1)) were from porcine blood, which nonetheless exhibits good agreement between the proposed non-Newtonian power-law viscosity model and human blood, as described in previous publications, with shear rates from 0.01 s^−1^ to 250 s^−1^ [2,3,33,34,35]. It has been found that porcine blood shares many common hemodynamic characteristics with human blood, i.e., RBCs [36,37,38,39], and similar shear-thinning properties [39,40]. However, some other research discovered some differences between human and porcine bloods in terms of shear-thinning properties, especially under some special conditions, e.g., lower shear rates [40]. The discrepancies between different research groups may stem from insufficient statistical analyses due to limited sample sizes. More comprehensive studies are needed to address these ambiguous conclusions.

### 2.2. Blood Analog Generation

#### 2.2.1. Materials

**Figure 5:**
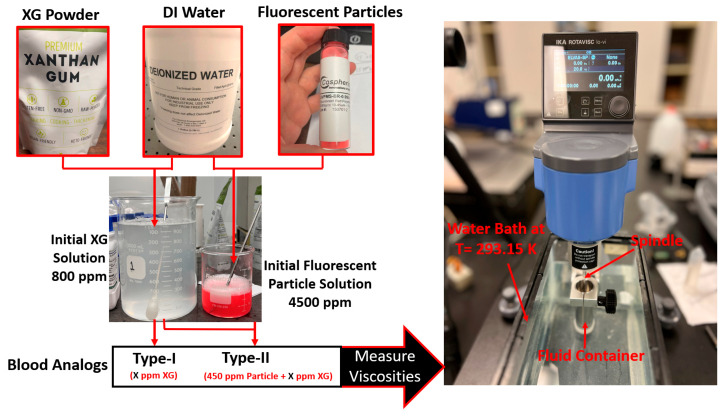
Blood analog workflow and viscosity measurements.

As shown in Figure 5, two synthetic blood analogues, i.e., Type I and Type II, were generated to replicate the shear-thinning properties of blood under the designated thermal conditions ranging from 295 to 312 K. Type I was intended for general use and consisted of DI water (supplied by the Chemistry Department at the Wright State University, Dayton, OH, USA) and xanthan gum (XG) powder (Kate Naturals, Harrisonburg, VA, USA). Type II was designed for use in PIV experiments to study hemodynamics in vessels or heart models, which require a transparent blood analog with seeded particles. The Type II analog was a mixture of DI water, XG power, and FRPM particles (Cospheric LLC, Somis, CA, USA), with particle sizes ranging from 10 to 45 μm.

To prepare blood analogs, the process began by mixing boiling DI water with XG powder to create the base XG solution, with a concentration of 800 ppm. This solution was then diluted with DI water to generate the desired concentrations for Type I blood analogs at specified thermal conditions. For Type II blood analogs, DI water and FRPM solution were added to the XG solution to achieve the designated concentrations and ensure that 450 ppm seeded FRPM particles were present for PIV experiments.

It is worth mentioning that the mixing of DI water and XG powder was a delicate and technically challenging process, as it required complete dissolution of XG powders without the formation of clumps, particles, or bubbles. In the mixing process, a magnetic stirrer was employed to ensure minimal temperature discrepancies in the solution, as well as the full dissolution of the XG powder in the DI water. To avoid the aggregation of XG powder, a stirring rod was also used manually at the moment of adding the XG powder to the boiled DI water. In addition, to ensure the consistency and accuracy of the blood analogs, three batches of original blood analogs were created. The viscosities of these batches were measured under shear rates ranging from 1 to 250 s^−1^, and the discrepancy was kept under 3%. In the preparation of the Type II blood analogs, a raw FRPM particle solution at 4500 ppm was prepared. Similar to the XG solution, three batches of the FRPM particle solution (i.e., 100 mL of each) were made, with differences among them remaining within 3%. It is worth mentioning that to ensure FRPM that the particles were fully suspended in the DI water, 0.1 mL detergent, as a surfactant, was added into the solution to reduce the surface tension of the liquid.

To create Type I blood analogs, the 800 ppm XG solution was diluted with DI water to form a 16 mL solution with the XG concentration ranging from 120 to 600 ppm, in increments of 20 ppm. For Type II blood analogs, the 800 ppm XG solution was diluted in the same manner to form an 18 mL solution, to which 2 mL of 4500 ppm FRPM solution was added, resulting in a total volume of 20 mL. Such a step was to ensure that all Type II blood analogs were uniformly seeded with the same quantity (450 ppm) of FRPM particles, satisfying the requirements for PIV tests to secure accurate flow characteristics in the tested vessel or heart model.

#### 2.2.2. Experimental Setup and Measurements

The prepared solutions were thoroughly mixed by stirring in a beaker (see Figure 5) and then allowed to stand for sufficient time to ensure the complete dissipation of any visible bubbles. Once the solution was bubble-free, it was transferred to a fluid container and placed in a custom water bath. The water bath maintained the blood analog at a stable environmental temperature of 293.15 K. Viscosity measurements were conducted using an IKA Rotavisc lo-vi viscometer (IKA-Werke GmbH & Co. KG, Staufen, Germany). Prior to testing, the stability of IKA Rotavisc lo-vi device was verified by rotating the bottom standers to ensure proper functionality.

During the viscosity measurement process, two independent operators performed the tests. Each operator recorded viscosity readings twice, and the average of these measurements was taken. To minimize random errors, the results from both operators were compared systematically. Discrepancies, if any, were addressed by additional experiments to ensure the accuracy and reliability of the data. For each blood analog of a given concentration, the measured viscosity data were compared with viscosity values calculated using Equation (1). This comparison was used to match the analog’s viscosity with that of human blood at a specific body temperature. For the matching process, temperatures from 292 to 320 K were applied in increments of 0.01 K for the best match by minimizing the root mean square deviation (RMSD), which was calculated by

(18) $$RMSD=\sqrt{\frac{\sum_{n=1}^{N}{\left(\mu_{exp.}-\mu_{equation\;(1)}\right)}^{2}}{N}}$$

where $\mu_{exp.}\;$is the experimental measured values, $\mu_{Equation\;(1)}$ is the viscosity calculated using Equation (1) under certain temperatures, and N is the number of data points. The averaged relative discrepancy $\bar{D}$ in the viscosity comparison between the developed blood analog and Equation (1) was calculated by

(19) $$\bar{D}=\frac{\sum_{n=1}^{N}\left|\frac{\mu_{exp.}-\mu_{Equation\;(1)}}{\mu_{Equation\;(1)}}\right|}{N}\times 100\%$$

## 3. Results

### 3.1. Type I Blood Analog

**Figure 6:**
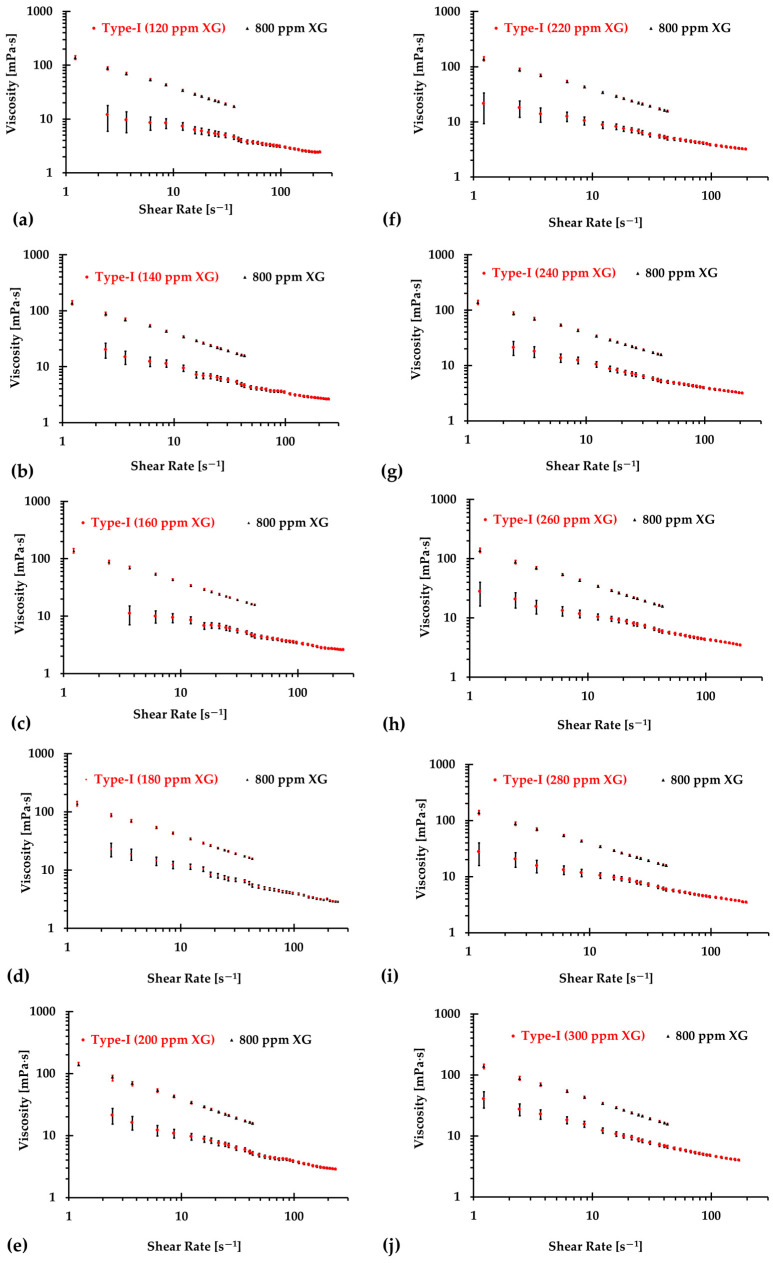
The relationship between shear rate and viscosity of Type I blood analogs under various XG concentrations: (**a**) 120 ppm; (**b**) 140 ppm; (**c**) 160 ppm; (**d**) 180 ppm; (**e**) 200 ppm; (**f**) 220 ppm; (**g**) 240 ppm; (**h**) 260 ppm; (**i**) 280 ppm; and (**j**) 300 ppm.

**Figure 7:**
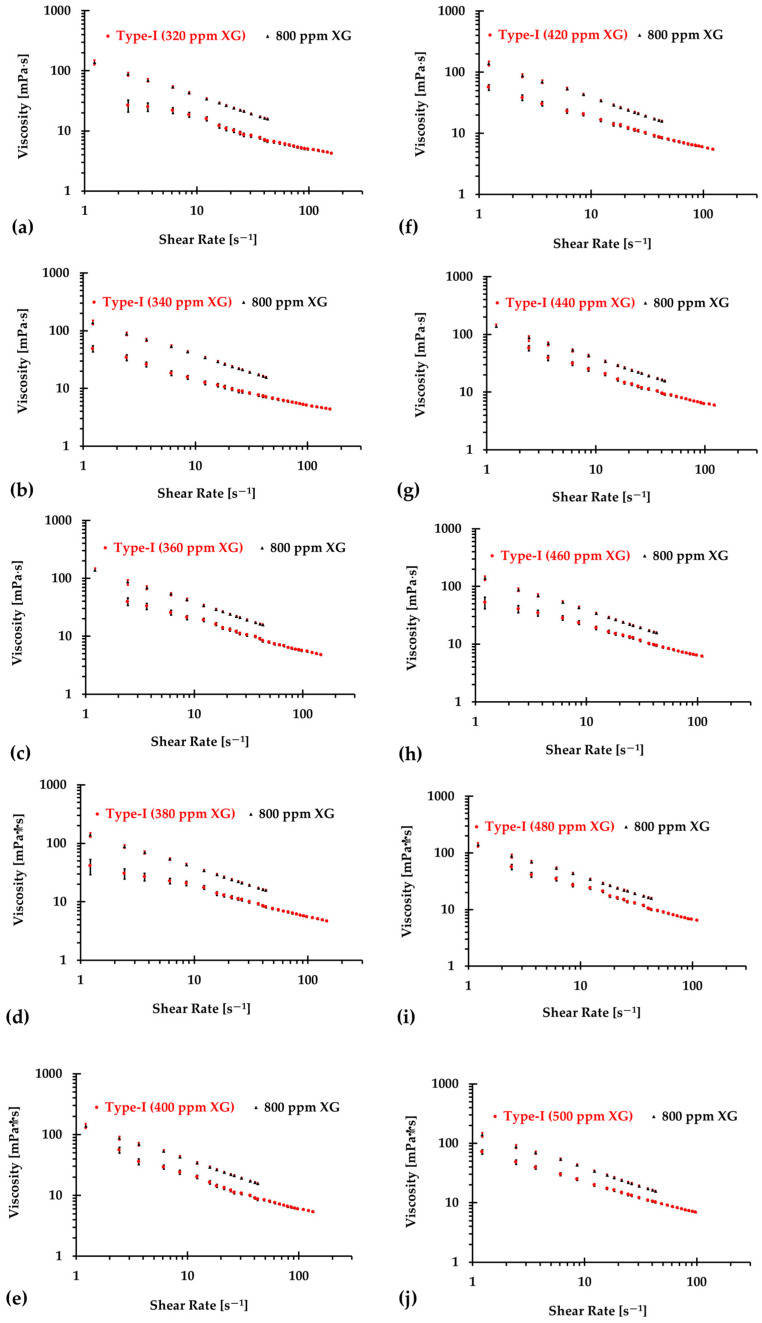
The relationship between shear rate and viscosity of Type I blood analogs under various XG concentrations (Continued): (**a**) 320 ppm; (**b**) 340 ppm; (**c**) 360 ppm; (**d**) 380 ppm; (**e**) 400 ppm; (**f**) 420 ppm; (**g**) 440 ppm; (**h**) 460 ppm; (**i**) 480 ppm; and (**j**) 500 ppm.

**Figure 8:**
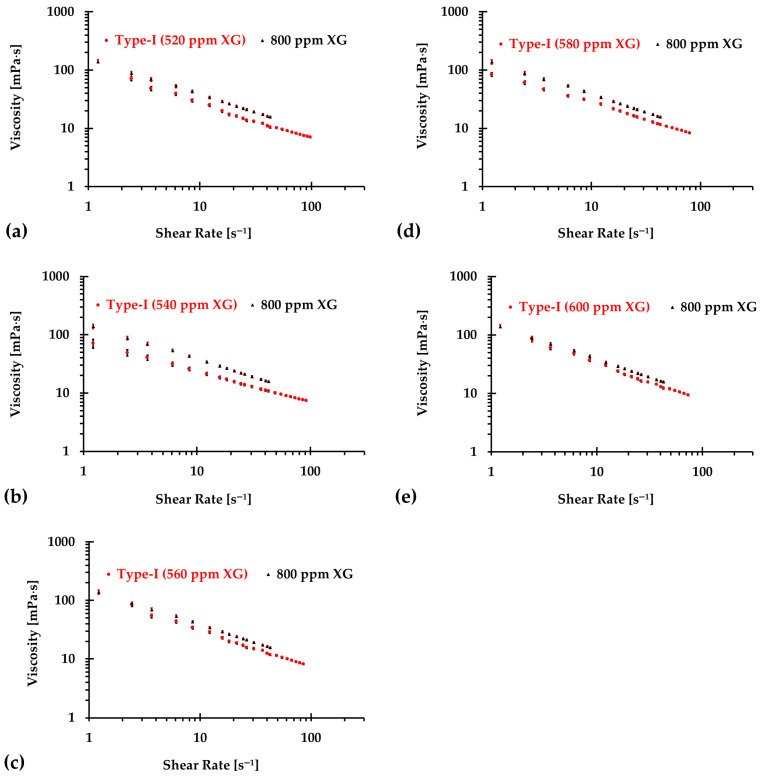
The relationship between shear rate and viscosity of Type I blood analogs under various XG concentrations (Continued): (**a**) 520 ppm; (**b**) 540 ppm; (**c**) 560 ppm; (**d**) 580 ppm; and (**e**) 600 ppm.

**Figure 9:**
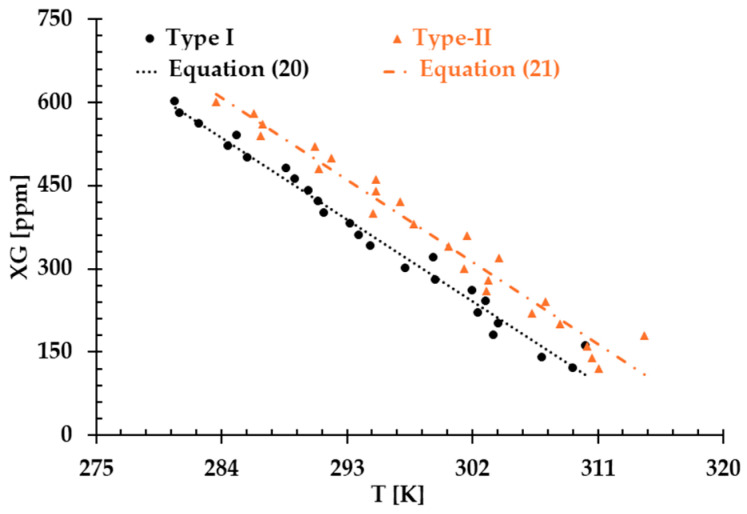
The relationship between temperature and XG concentration in the blood analog.

The results presented in Figure 6, Figure 7 and Figure 8 illustrate the relationship between shear rates and viscosity in Type I blood analogs, with XG concentrations varying in increments of 20 ppm. These measurements were taken at a constant room temperature of 293.15 K, with the original base solution of 800 ppm XG serving as a reference. The data demonstrate that all generated Type I blood analogs exhibited shear-thinning rheological properties similar to those of real blood, where viscosity decreases as the shear rate increases. By minimizing RSMD between experimental measurements and the developed Equation (1), we derived the relationship between temperature and XG concentration for Type I blood analogs, as shown in Figure 9. The results revealed a strong linear correlation between temperature and XG concentration $c_{XG}$ ppm, with a registered coefficient of determination $R^{2}=0.9828$ and $\bar{D}$ < 5%. Specifically, this relationship can be expressed by the following equation:

(20) $$c_{XG}\;[ppm]=-16.317T+5170.10,T\;[K]\in [295,312]$$

Equation (20) provides a formula to generate blood analogs for in vitro experiments that require a realistic shear-thinning rheological environment, simulating blood flow behavior at various “human body” temperatures, i.e., from 295 to 312 K.

### 3.2. Type II Blood Analog

**Figure 10:**
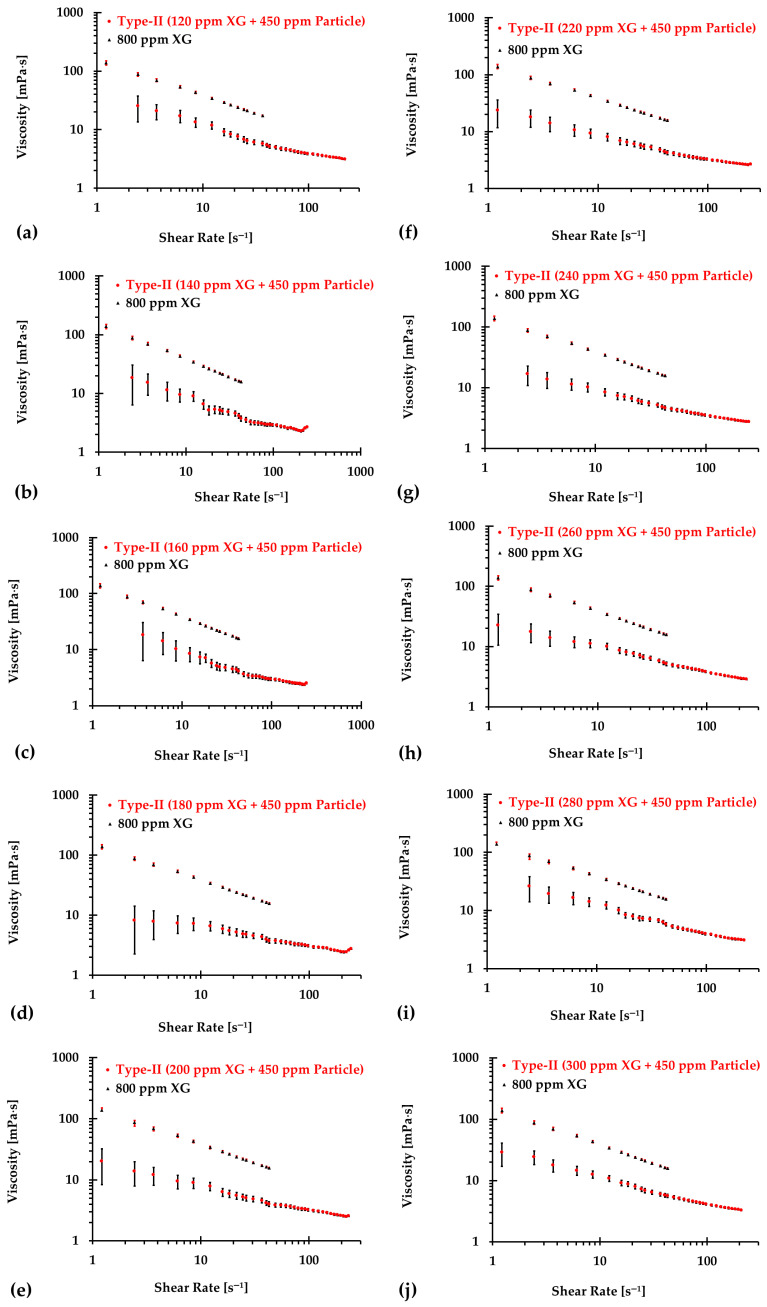
The relationship between shear rate and viscosity of Type II blood analogs under various concentrations: (**a**) 120 ppm XG and 450 ppm particle; (**b**) 140 ppm XG and 450 ppm particle; (**c**) 160 ppm XG and 450 ppm particle; (**d**) 180 ppm XG and 450 ppm particle; (**e**) 200 ppm XG and 450 ppm particle; (**f**) 220 ppm XG and 450 ppm particle; (**g**) 240 ppm XG and 450 ppm particle; (**h**) 260 ppm XG and 450 ppm particle; (**i**) 280 ppm XG and 450 ppm particle; and (**j**) 300 ppm XG and 450 ppm particle.

**Figure 11:**
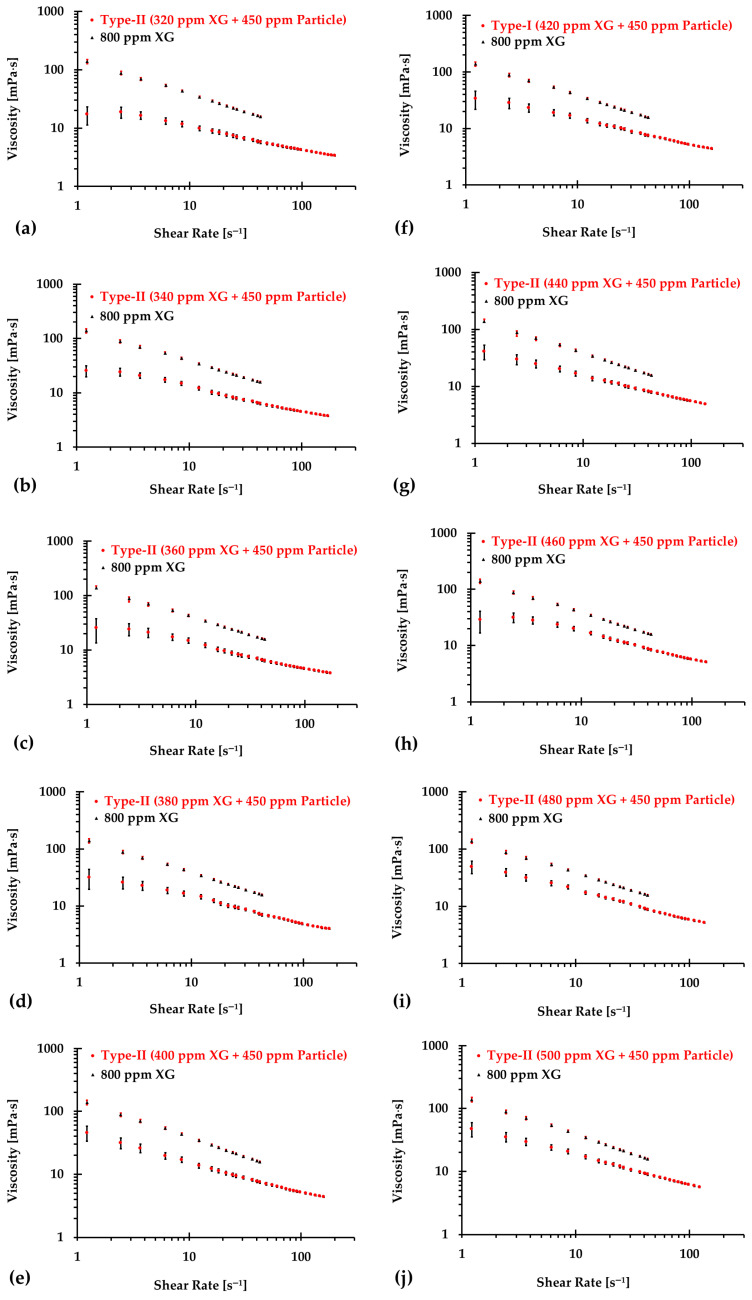
The relationship between shear rate and viscosity of Type II blood analogs under various concentrations (continued): (**a**) 320 ppm XG and 450 ppm particle; (**b**) 340 ppm XG and 450 ppm particle; (**c**) 360 ppm XG and 450 ppm particle; (**d**) 380 ppm XG and 450 ppm particle; (**e**) 400 ppm XG and 450 ppm particle; (**f**) 420 ppm XG and 450 ppm particle; (**g**) 440 ppm XG and 450 ppm particle; (**h**) 460 ppm XG and 450 ppm particle; (**i**) 480 ppm XG and 450 ppm particle; and (**j**) 500 ppm XG and 450 ppm particle.

**Figure 12:**
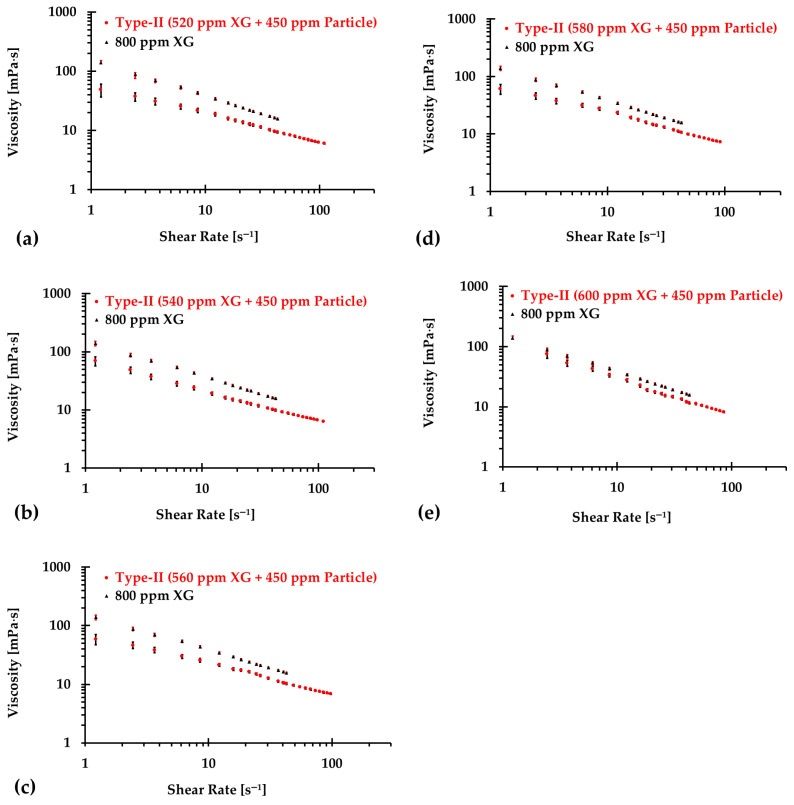
The relationship between shear rate and viscosity of Type II blood analogs under various concentrations (continued): (**a**) 520 ppm XG and 450 ppm particle; (**b**) 540 ppm XG and 450 ppm particle; (**c**) 560 ppm XG and 450 ppm particle; (**d**) 580 ppm XG and 450 ppm particle; and (**e**) 600 ppm XG and 450 ppm particle.

Similarly, Figure 10, Figure 11 and Figure 12 present the experimental results of the measured viscosities of the generated Type II blood analogs across various shear rates, with XG concentrations varying in increments of 20 ppm. These measurements were conducted at a controlled room temperature of 293.15 K, and the raw solution used for Type II blood analogs had the same XG concentration of 800 ppm as the Type I blood analogs. The FRPM particle concentration in the Type II blood analogs was maintained at 450 ppm, ensuring consistency in PIV tests by maintaining a uniform particle concentration to avoid errors due to varied particle density in captured images. As shown in Figure 10, Figure 11 and Figure 12, the results demonstrate that all generated Type II blood analogs exhibited the shear-thinning rheological properties of real blood, where the viscosity decreases as shear rate increases. By minimizing RSMD between experimental measurements and the developed Equation (1), it was found that XG concentration $c_{XG}$ was linearly related to human-body temperature. This relationship is highlighted by an orange line in Figure 9, with $R^{2}=0.9642$ and $\bar{D}$ < 5%. Specifically, the relationship can be expressed by the following equation:

(21) $$c_{XG}\;[ppm]=-16.317T+5170.10,T\;[K]\in [295,312]$$

Equation (21) provides a formula to generate Type II blood analogs which replicate the shear-thinning properties of real blood and ensure accurate hemodynamic patterns in in-vitro neurovascular and cardiovascular models specifically for PIV tests.

## 4. Discussion

The results presented above demonstrate that both Type I and Type II blood analogs exhibited a linear relationship between XG concentration and body temperature. As temperature decreases, the internal resistance of the blood increases due to the rise in viscosity [5]. Additionally, as shown in Figure 9, at the same temperature, the XG concentration of the Type II blood analog was higher than that of the Type I blood analog. This difference was attributed to the addition of 450 ppm of FRPM particle solution, which further diluted the XG solution, requiring a higher XG concentration to ensure that the viscosity of Type II blood analogs matched the viscosity of the corresponding Type I blood analogs.

**Figure 13:**
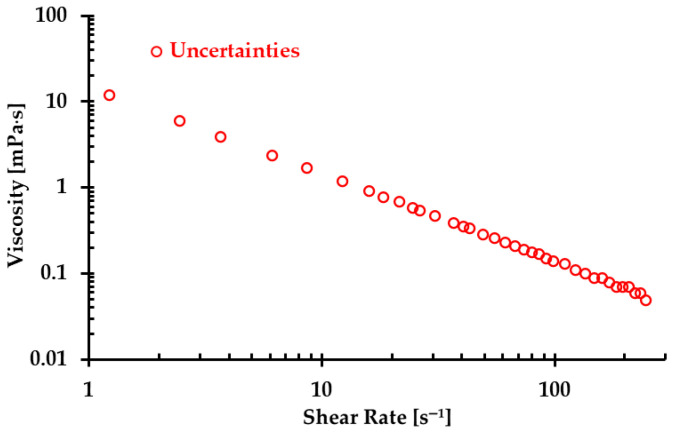
The maximum uncertainty in viscosity under the corresponding shear rate due to errors in the viscometer measurements.

Despite our team’s best efforts to minimize the errors and uncertainties during the measurements, discrepancies were still observed in the formulas in the two types of blood analog under varying thermal conditions, as depicted in Figure 9. These discrepancies were primarily attributed to the random errors caused by systematic uncertainties in the viscometer, especially for low shear rates, as illustrated in Figure 13. It was found that although the uncertainty decreased with the increasing shear rate, it remained significant with shear rates from 1.22 to 12.22 s^−1^. In this range, the possible uncertainties in viscosity measurements ranged from 1.19 to 12 mPa·s. For instance, for one blood analog with 120 ppm XG and 450 ppm particles, we measured an average viscosity of 25.5 mPa·s at a shear rate of 1.22 s^−1^. At this shear rate, the systematic uncertainty was 12 mPa·s, which may have substantially impacted the accuracy of the formulas used to generate blood analogs at the designated temperatures.

**Figure 14:**
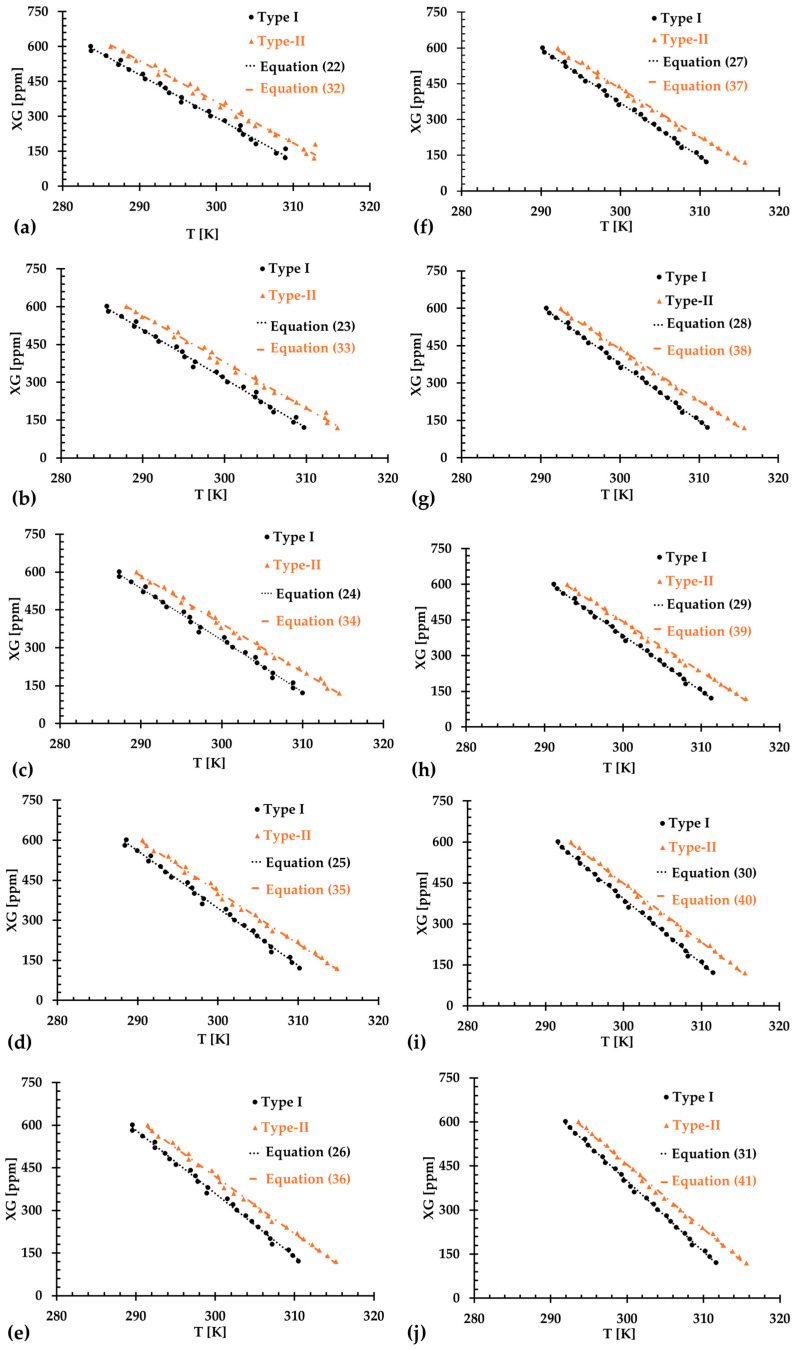
The relationship between XG concentration and temperature in the generated blood analogs under various numbers of skipped initial data points: (**a**) 1 data point; (**b**) 2 data points; (**c**) 3 data points; (**d**) 4 data points; (**e**) 5 data points; (**f**) 6 data points; (**g**) 7 data points; (**h**) 8 data points; (**i**) 9 data points; and (**j**) 10 data points.

To reduce uncertainties for the regression models, we made efforts to improve the agreement by omitting the first few data points, specifically ranging from 1 to 10. Figure 14a–j display the relationship between temperature and XG concentration for both Type I and Type II blood analogs, with designated initial data points being skipped. The linearly regressed models for each situation are given in Equations (22)–(31) and Equations (32)–(41) for Type I and Type II blood analogs, respectively.

(22–31) $$c_{XG}\;[ppm]=-\left[\begin{array}{c} 17.615\\ 18.292\\ 19.015\\ 19.635\\ 20.116\\ 20.427\\ 20.782\\ 21.173\\ 21.568\\ 21.918 \end{array}\right]T+\left[\begin{array}{c} 5647.0\\ 5867.4\\ 6099.7\\ 6298.4\\ 6455.0\\ 6557.7\\ 6670.8\\ 6794.5\\ 6919.5\\ 7030.7 \end{array}\right],T\;[K]\in [290,315],\;\mathrm{Type}\;I$$

(32–41) $$c_{XG}\;[ppm]=-\left[\begin{array}{c} 18.372\\ 19.426\\ 20.576\\ 21.513\\ 22.207\\ 22.654\\ 23.080\\ 23.477\\ 23.829\\ 24.148 \end{array}\right]T+\left[\begin{array}{c} 5806.1\\ 6141.3\\ 6503.3\\ 6798.6\\ 7021.4\\ 7166.4\\ 7301.9\\ 7428.5\\ 7541.3\\ 7643.9 \end{array}\right],T\;\left[K\right]\in \left[290,315\right],\;\mathrm{Type}\;\mathrm{II}$$

For Type I blood analogs, the $R^{2}$ of the regressed Equations (22)–(31) were registered as 0.9888, 0.9941, 0.9955, 0.9959, 0.9963, 0.9963, 0.9967, 0.9970, 0.9972, and 0.9974, respectively, for datasets where the number of 1 to 10 initial data points were skipped. For the Type II blood analog, the $R^{2}$ for the corresponding Equations (32)–(41) were registered as 0.9926, 0.9940, 0.9939, 0.9946, 0.9957, 0.9974, 0.9976, 0.9976, 0.9978, and 0.9980, respectively, when the number of 1 to 10 initial data points were excluded correspondingly. The best fit was achieved by excluding the first 10 data points at lower low shear rates. These initial data often exhibited steep changes and large uncertainties, which could have otherwise impacted the accuracy of the regression models.

**Figure 15:**
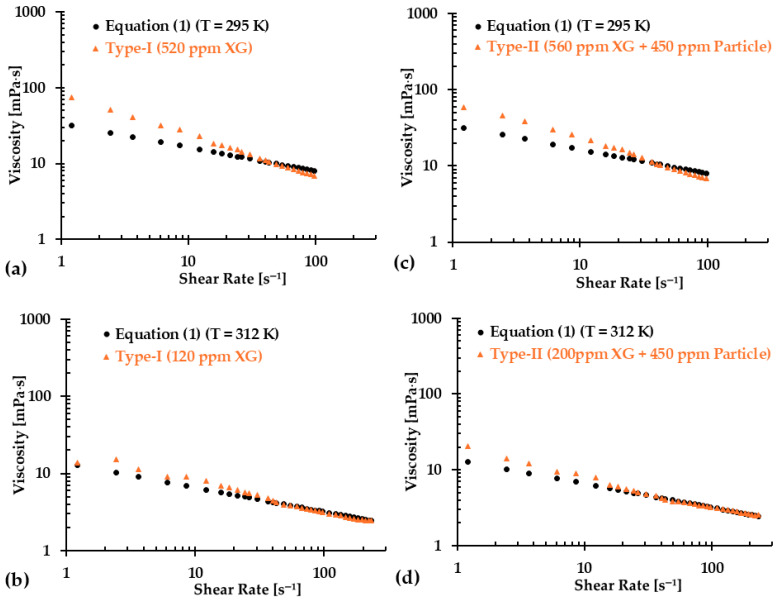
Comparisons in viscosity between the regression equations and Equation (1) at two representative temperatures for the two types of blood analogs: (**a**) Type I at T = 295 K, (**b**) Type I at T = 312 K, (**c**) Type II at T = 295 K, and (**d**) Type II at T = 312 K.

However, it is important to note that while skipping more data points resulted in better regressed equations, as indicated by the $R^{2}$ values, over-fittings can introduce new challenges. Figure 15a–d illustrate the comparison between regression equations and the baseline Equation (1) for both blood analog types at two representative temperatures (i.e., T = 295 K and 312 K), with the first 10 data points having been excluded. The results show that for a shear rate greater than 30 s^−1^, there was a good match between the regression equations and the baseline Equation (1). However, noticeable discrepancies appeared in the viscosity values for shear rates ranging from 0 to 30 s^−1^. As more initial points were skipped, the discrepancies in the viscosity values at low shear rates became more pronounced. These differences could be attributed to the intrinsic physical properties of the XG solution. By skipping fewer initial data points, a better match could be achieved in the viscosity comparison at shear rates of 0–30 s^−1^. However, the difference became more significant at higher shear rate, such as greater than 30 s^−1^. Therefore, we recommend that researchers first identify the shear rate range that is relevant to their experiments and then select the appropriate regression formulas (i.e., Equations (20)–(41)) to generate the desired blood analogs for their specific applications.

## 5. Conclusions

In this study, we presented a synthetic method for developing a non-Newtonian human blood model (Equation (1)) that explicitly captures shear-thinning properties as a function of temperature, based on real blood viscosities. Using Equation (1) as the baseline, we successfully generated two types of blood analogs, i.e., Type I and Type II, for different purposes, across a temperature range of 295–312 K, with both exhibiting the same shear-thinning rheological properties. The Type I blood analog, composed solely of XG solution, was designed for general use, while the Type II blood analog, a mixture of XG solution and FRMP particles, was developed for PIV measurements to capture hemodynamic characteristics in in vitro cardiovascular and neurovascular models. Both blood analogs demonstrated a linear relationship between XG concentration and body temperature. Although omitting the initial few data points improved the matching performance of the regression models at high shear rate (>30 s^−1^), this approach introduced potential errors in terms of predicting viscosities at lower shear rate (<30 s^−1^). Therefore, it is crucial for researchers to select appropriate formulas based on their experimental shear rate range to ensure accurate blood analog generation.

## 6. Limitations and Future Work

This study did not simulate blood cells explicitly. Although FRMP particles in the Type II blood analog partially represent blood cells, their concentration of 450 ppm was much lower than the red blood cell (RCB) concentrations in humans, which ranges 4.7 to 6.1 million ppm for males, 4.2 to 5.4 million ppm for females, and 4.0 to 5.5 million ppm for children [41]. Future studies could incorporate alternative materials, such as polysaccharide particles, to mimic blood cells more accurately by considering the effects of hematocrit changes with a wider range. Nevertheless, the solid-like elastic properties of blood and its transient rheology were not considered in this study, mainly due to the limitation of the current instruments.

More specifically, the viscosity fluctuation in transient rheology was mainly due to the aggregation and disaggregation of RCBs at low shear rates [1,3,27]. The current study focused on developing blood analogs (i.e., hematocrit of 40%, plasma, red blood cell deformability) to mimic steady-state non-Newtonian shear-thinning viscosities within a shear rate range of 1–250 s^−1^, while the contribution of the aggregation and disaggregation of RCBs at low shear rates (specifically smaller than 1 s^−1^) to the viscosity was not explicitly considered. Our future research on blood analog generation will take these limitations into consideration more systematically. This would allow for more realistic blood analog generation and enable further investigations of the hemodynamic behaviors associated with the pathophysiology of neurovascular and cardiovascular diseases.

Furthermore, we did not quantify the storage duration of the blood analogs. In our previous experiments [5,42], we found that the blood analog viscosities were extremely close at the corresponding shear rates under a time gap of over one month. However, the exact storage duration under various environmental temperatures needs to be measured specifically in the future study.

## Figures and Tables

**Table 1:** Using shear rate and temperature increments to determine the temperature index constant α.

∆T	$\dot{\gamma }\;[s^{-1}]$						
	6.11	12.22	24.45	36.68	48.91	61.15	73.37
	α [K]						
$∆T_{1-2}$	5078.0695	5076.4238	5642.7484	5753.1148	5735.2348	5484.2840	4880.5709
$∆T_{1-3}$	5075.0346	5001.5716	5275.4079	5372.0900	5392.6819	5288.4887	5083.2582
$∆T_{1-4}$	4914.6750	4815.3147	4961.1219	5035.4353	5004.6332	4936.2186	4811.9285
$∆T_{2-3}$	5072.6236	4942.1073	4983.5841	5069.3949	5120.5499	5132.9445	5244.2778
$∆T_{2-4}$	4845.6259	4704.9722	4673.0723	4732.1500	4695.8871	4704.6106	4782.9208
$∆T_{3-4}$	4587.6398	4435.4649	4320.1714	4348.8666	4213.2520	4217.8034	4258.5822

**Table 2:** Calculated $T_{Ref.}$ under four designated temperatures.

T [K]	$\dot{\gamma }\;[s^{-1}]$						
	6.11	12.22	24.45	36.68	48.91	61.15	73.37
	$T_{Ref.}\;[K]$						
295.5	295.70.1	295.5	295.70.1	295.5	295.70.1	295.5	295.70.1
300.2	298.847	300.2	298.847	300.2	298.847	300.2	298.847
305.8	307.289	305.8	307.289	305.8	307.289	305.8	307.289
310.3	314.587	310.3	314.587	310.3	314.587	310.3	314.587

## Data Availability

Data available on request due to restrictions, e.g., privacy or ethics.

## Abbreviations

The following abbreviations are used in this manuscript:

DI	Deionized
FRPM	Fluorescent red polyethylene microspheres
PIV	Particle image velocimetry
RCB	Red blood cell
RMSD	Root mean square deviation
TEVP	Thixo-elasto-visco-plastic
XG	Xanthan gum

## References

[1] Armstrong M., Horner J., Clark M., Deegan M., Hill T., Keith C., Mooradian L. (2018). Evaluating rheological models for human blood using steady state, transient, and oscillatory shear predictions. Rheol. Acta.

[2] Merrill E.W., Cokelet G.C., Britten A., Wells R.E. (1963). Non-Newtonian Rheology of Human Blood: Effect of Fibrinogen Deduced by "Subtraction". Circ. Res..

[3] Nader E., Skinner S., Romana M., Fort R., Lemonne N., Guillot N., Gauthier A., Antoine-Jonville S., Renoux C., Hardy-Dessources M.-D. (2019). Blood Rheology: Key Parameters, Impact on Blood Flow, Role in Sickle Cell Disease and Effects of Exercise. Front. Physiol..

[4] Lynch S., Nama N., Figueroa C.A. (2022). Effects of non-Newtonian viscosity on arterial and venous flow and transport. Sci. Rep..

[5] Yi H., Yang Z., Johnson M., Bramlage L., Ludwig B. (2022). Hemodynamic characteristics in a cerebral aneurysm model using non-Newtonian blood analogues. Phys. Fluids.

[6] Ekici M., Çakır Biçer N., Yirün A., Demirel G., Erkekoğlu P. (2024). Evaluation of Exposure to Bisphenol Analogs Through Canned and Ready-to-Eat Meal Consumption and Their Possible Effects on Blood Pressure and Heart Rate. Nutrients.

[7] Basson N., Peng C.-H.S., Geoghegan P., van der Lecq T., Steven D., Williams S., Lim A.E., Ho W.H. (2024). A computational fluid dynamics investigation of endothelial cell damage from glaucoma drainage devices. Sci. Rep..

[8] Knüppel F., Thomas I., Wurm F.-H., Torner B. (2023). Suitability of Different Blood-Analogous Fluids in Determining the Pump Characteristics of a Ventricular Assist Device. Fluids.

[9] Froese V., Gabel G., Parnell J., Prause A., Lommel M., Kertzscher U. (2022). Flow study on a transparent two-phase blood model fluid based on alginate microspheres. Exp. Fluids.

[10] Walker A., Johnston C., Rival D. (2014). On the Characterization of a Non-Newtonian Blood Analog and its Response to Pulsatile Flow Downstream of a Simplified Stenosis. Ann. Biomed. Eng..

[11] Kalke B.R., Mantini E.L., Kaster R.L., Carlson R.G., Lillehei C.W. (1967). Hemodynamic features of a double-leaflet prosthetic heart valve of new design. ASAIO J..

[12] Ling S.C., Atabek H.B., Fry D.L., Patel D.J., Janicki J.S. (1968). Application of Heated-Film Velocity and Shear Probes to Hemodynamic Studies. Circ. Res..

[13] Baldwin J.T., Deutsch S., Geselowitz D.B., Tarbell J.M. (1994). LDA Measurements of Mean Velocity and Reynolds Stress Fields Within an Artificial Heart Ventricle. J. Biomech. Eng..

[14] Kempainen R.R., Brunette D.D. (2004). The evaluation and management of accidental hypothermia. Respir. Care.

[15] Lell B., Brandts C.H., Graninger W., Kremsner P.G. (2000). The circadian rhythm of body temperature is preserved during malarial fever. Wien Klin Wochenschr.

[16] Bouchama A., Knochel J.P. (2002). Heat Stroke. N. Engl. J. Med..

[17] Sarkar M., Prabhu V. (2017). Basics of cardiopulmonary bypass. Indian J Anaesth..

[18] Jaiswal A.N., Vagga A. (2022). Cryopreservation: A Review Article. Cureus.

[19] Choi Y., Jung S.L. (2020). Efficacy and Safety of Thermal Ablation Techniques for the Treatment of Primary Papillary Thyroid Microcarcinoma: A Systematic Review and Meta-Analysis. Thyroid.

[20] Najjari M.R., Hinke J.A., Bulusu K.V., Plesniak M.W. (2016). On the rheology of refractive-index-matched, non-Newtonian blood-analog fluids for PIV experiments. Exp. Fluids.

[21] Yousif M.Y., Holdsworth D.W., Poepping T.L. (2011). A blood-mimicking fluid for particle image velocimetry with silicone vascular models. Exp. Fluids.

[22] Bai K., Katz J. (2014). On the refractive index of sodium iodide solutions for index matching in PIV. Exp. Fluids.

[23] Brookshier K.A., Tarbell J.M. (1993). Evaluation of a transparent blood analog fluid: Aqueous Xanthan gum/glycerin. Biorheology.

[24] Stephanova D.I., Kossev A. (2018). Theoretical predication of temperature effects at 20–42 °C on adaptive processes in simulated amyotrophic lateral sclerosis. JIN.

[25] Mann D.E., Tarbell J.M. (1990). Flow of non-Newtonian blood analog fluids in rigid curved and straight artery models. Biorheology.

[26] Naiki T. (1995). Evaluation of High Polymer Solutions as Blood Analog Fluid-For the Model Study of Hemodynamics. J. Jpn. Soc. Biorheology.

[27] Kaliviotis E., Yianneskis M. (2011). Blood viscosity modelling: Influence of aggregate network dynamics under transient conditions. Biorheology.

[28] Kannojiya V., Das A.K., Das P.K. (2021). Simulation of Blood as Fluid: A Review from Rheological Aspects. IEEE Rev. Biomed. Eng..

[29] Gutmann F., Simmons L.M. (1952). The Temperature Dependence of the Viscosity of Liquids. J. Appl. Phys..

[30] Tajima Y.A., Crozier D.G. (1986). Chemorheology of an amine-cured epoxy resin. Polym. Eng. Sci..

[31] Roller M.B. (1986). Rheology of curing thermosets: A review. Polym. Eng. Sci..

[32] Tanner R.I. (2000). Engineering Rheology.

[33] Chien S., Usami S., Taylor H.M., Lundberg J.L., Gregersen M.I. (1966). Effects of hematocrit and plasma proteins on human blood rheology at low shear rates. J. Appl. Physiol..

[34] Skalak R., Keller S.R., Secomb T.W. (1981). ASME Centennial Historical Perspective Paper: Mechanics of Blood Flow. J. Biomech. Eng..

[35] Suzuki T., Takao H., Suzuki T., Suzuki T., Masuda S., Dahmani C., Watanabe M., Mamori H., Ishibashi T., Yamamoto H. (2017). Variability of hemodynamic parameters using the common viscosity assumption in a computational fluid dynamics analysis of intracranial aneurysms. Technol. Health Care.

[36] Pond W.G., Houpt K.A. (1978). The Biology of the Pig.

[37] Jandl J.H. (1987). Blood: Textbook of Hematology.

[38] Sondeen J.L., de Guzman R., Amy Polykratis I., Dale Prince M., Hernandez O., Cap A.P., Dubick M.A. (2013). Comparison between human and porcine thromboelastograph parameters in response to ex vivo changes to platelets, plasma, and red blood cells. Blood Coagul. Fibrinolysis.

[39] Laurent A., Durussel J.J., Dufaux J., Penhouët L., Bailly A.L., Bonneau M., Merland J.J. (1999). Effects of contrast media on blood rheology: Comparison in humans, pigs, and sheep. Cardiovasc. Interv. Radiol..

[40] Ecker P., Sparer A., Lukitsch B., Elenkov M., Seltenhammer M., Crevenna R., Gföhler M., Harasek M., Windberger U. (2021). Animal blood in translational research: How to adjust animal blood viscosity to the human standard. Physiol. Rep..

[41] Kibble J.D. (2020). The Big Picture Physiology: Medical Course & Step 1 Review.

[42] Yi H., Yang Z., Johnson M., Bramlage L., Ludwig B. (2022). Developing an in vitro validated 3D in silico internal carotid artery sidewall aneurysm model. Front. Physiol..

